# Clinical analysis of Kimura’s disease in 24 cases from China

**DOI:** 10.1186/s12893-019-0673-7

**Published:** 2020-01-02

**Authors:** Guoliang Zhang, Xumao Li, Guangbin Sun, Yitan Cao, Nan Gao, Weidong Qi

**Affiliations:** Department of Otorhinolaryngology, Affiliated Huashan Hospital, Fudan University, Shanghai, 200040 China

**Keywords:** Kimura’s disease, Eosinophilia, Histopathology, Treatment

## Abstract

**Background:**

We reviewed details of Chinese Kimura’s disease (KD) cases. A full clinical analysis was subsequently performed to improve the accuracy of clinical diagnosis and treatment of KD.

**Methods:**

A total of 24 patients with pathologically confirmed KD treated between March 2008 and March 2018 were reviewed retrospectively for clinical and histopathological analysis.

**Results:**

In the 24 KD cases, 20 were male and 4 were female with the age of onset ranging from 5 to 65 years. Lesion diameter ranged from 0.6 cm to 7 cm with unilateral involvement being more popular (79%). Imaging examination had a high detection rate for KD involving the parotid gland and subcutaneous but had low specificity. Microscopic analysis indicated that KD mainly involved subcutaneous soft tissue and lymph nodes. The prominent feature of lymphoid tissue was germinal center hyperplasia surrounded by several lobules associated with hyperplastic vascular structures. Out of the 24 patients, 11 experienced recurrence of disease after treatment (surgical resection: 46.2%, surgical resection followed by oral corticosteroids: 71.4% and surgical resection combined with radiotherapy: 0%).

**Conclusions:**

Our analysis revealed clinical, imaging, and histological characteristics of KD. A better understanding of the disease will help clinicians reduce misdiagnosis and improve the diagnostic rate upon patient first clinical visit.

## Background

Kimura’s disease (KD) is a rare, benign disorder associated with chronic inflammatory lesions of unclear etiology. It most commonly presents in the head and neck region of Asian males during their second and fourth decades of life. However, some sporadic cases have been reported in Europe and America [1, 2]. Clinical examination generally reveals nontender subcutaneous swelling or nodules in the head and neck region that is partially associated with regional lymphadenopathy [3]. This disease is difficult to diagnose because of its resemblance to benign or malignant diseases and low morbidity.

Recently, several emerging studies have reported on the clinical characteristics of KD. Syed et al. showed detailed diffusion weighted imaging in KD, distinguishing it from other malignancies [4]. Additionally, two groups simultaneously identified angiolymphoid hyperplasia in KD case reports [5, 6]. Furthermore, in some cases, eosinophilia and multiple lymphadenopathy have consistently been found to be partly associated with KD occurrence [7]. While some histological identifications help characterize KD, more clinical features of this disease are needed for a differential diagnosis. To provide further insights into this disease, we analyzed detailed clinical data from a number of KD cases.

## Methods

### Patients

A total of 24 patients with pathologically confirmed KD were reviewed retrospectively. These patients were treated in our hospital from March 2008 to March 2018. Of these 24 patients, 22 were misdiagnosed at their first clinical visit. Detailed clinical data in our analysis included age, gender, laboratory workup, imaging, pathological diagnosis, treatments, and therapeutic results.

### Clinical managements

Three different modalities were applied to treat the 24 KD patients: Surgical resection alone (SE), surgical resection followed by oral corticosteroids (SE + OC), and surgical resection combined with radiotherapy (SE + R). Oral corticosteroids: prednisolone, 30 mg per day and the dosage was reduced gradually (30 mg × 5 d, 10 mg × 3 d, and 5 mg × 3 d). Radiotherapy: 36–45 Gy with 2 Gy per fraction.

### Treatment window

Patients were followed up until September 2018. Duration of follow-up ranged from 6 months to 113 months with a mean length of 55 months. Recurrence was determined through clinical, radiological examination, and histopathology.

### Statistical analysis

Chi-square test and Fisher’s exact test were used to compare the different treatment modality groups. All statistical analyses were completed using SPSS 23.0 (IBM Corp, Armonk, NY, USA) and a *P*-value < 0.05 was considered statistically significant.

## Results

### Clinical presentation

**Fig. 1:**
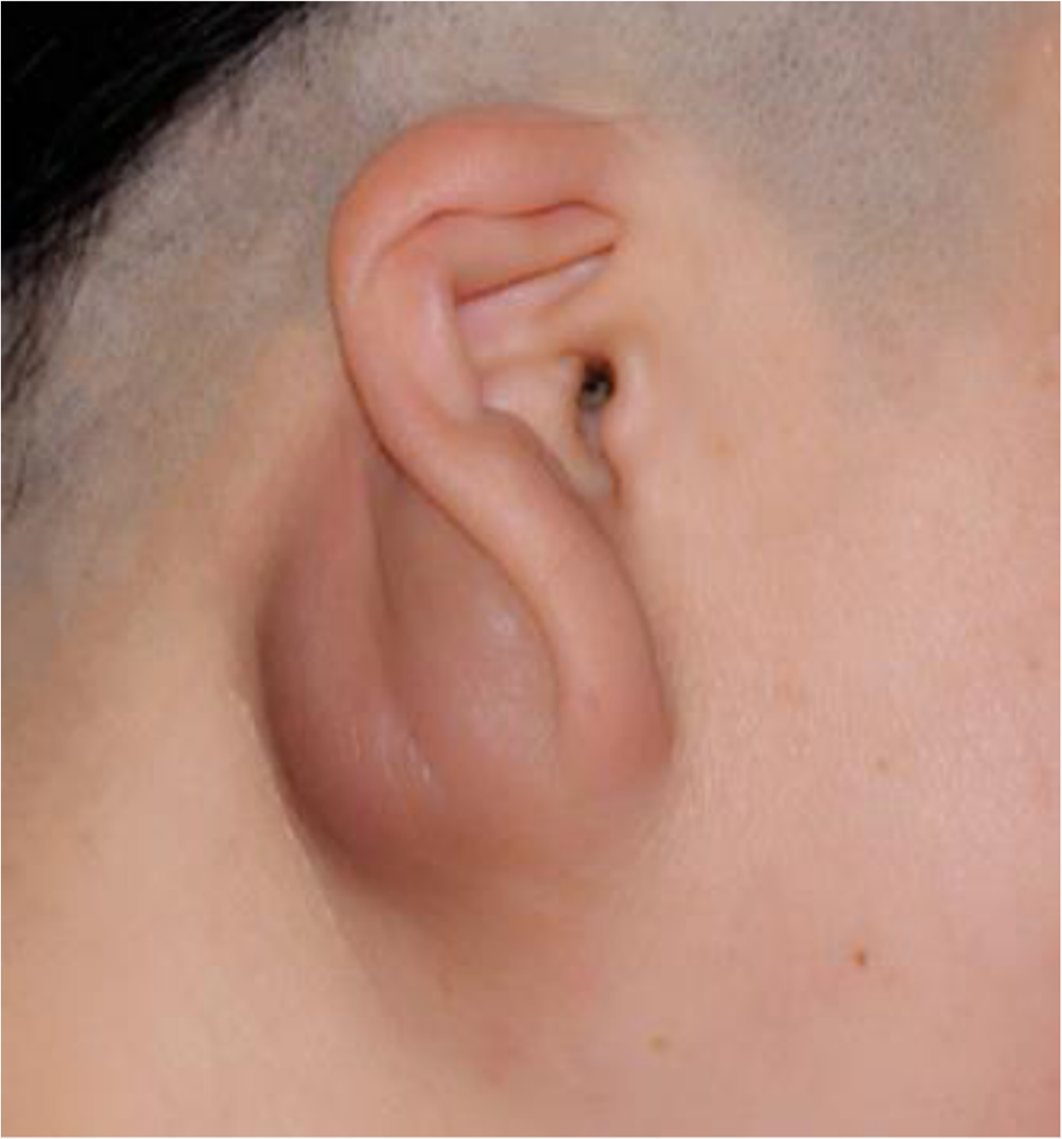
Kimura’s disease: The patient presented with unilateral parotid subcutaneous swelling. The overlying skin was accompanied by melanin pigmentation in lateral view

In the 24 KD cases, 20 were male and 4 were female. The age of onset ranged from 5 to 65 years with a median age of 44.5 years. Duration of symptoms ranged from 2 months to 17 years with a mean of 51.7 months. In all patients, the initial manifestation included painless subcutaneous nodules or focal swelling. With regard to anatomical distribution, most patients (71%) presented with focal lesions in the head and neck regions. The parotid region was the most frequently affected site (*n* = 10; Fig. 1). Unilateral involvement (79%) was more common than bilateral disease (21%). A total of 19 patients (79%) presented a single solitary lesion and 5 patients (21%) presented multiple lesions (Table 1). The main physical findings were irregular masses with pruritus (*n* = 8, 33.3%), melanin pigmentation (*n* = 5, 20.8%), and coarseness of overlying skin (*n* = 3, 12.5%). Increase in blood eosinophils were identified in 21 patients (87.5%), ranging from 11 to 51% compared to the normal range of 5%. Immunoglobulin examination was performed in 5 cases, all with elevated IgE levels.

**Table 1:** Clinical features of 24 patients with Kimura’s disease

Characteristics	No. of patients (%)
Gender	
Male	20(75.0%)
Female	4(25.0%)
Age at presentation	
< 35 years	3(12.5%)
≥ 35 years	21(87.5%)
Multiplicity	
Single	19(79.2%)
Multiple	5(20.8%)
Tumor diameter	
< 3 cm	7(29.2%)
≥ 3 cm	17(70.8%)
Location	
Head and neck region	17(70.8%)
Others	7(29.2%)
Peripheral blood eosinophil percentages	
Elevated	21(87.5%)
Normal	3(12.5%)

### Imaging analysis

**Fig. 2:**
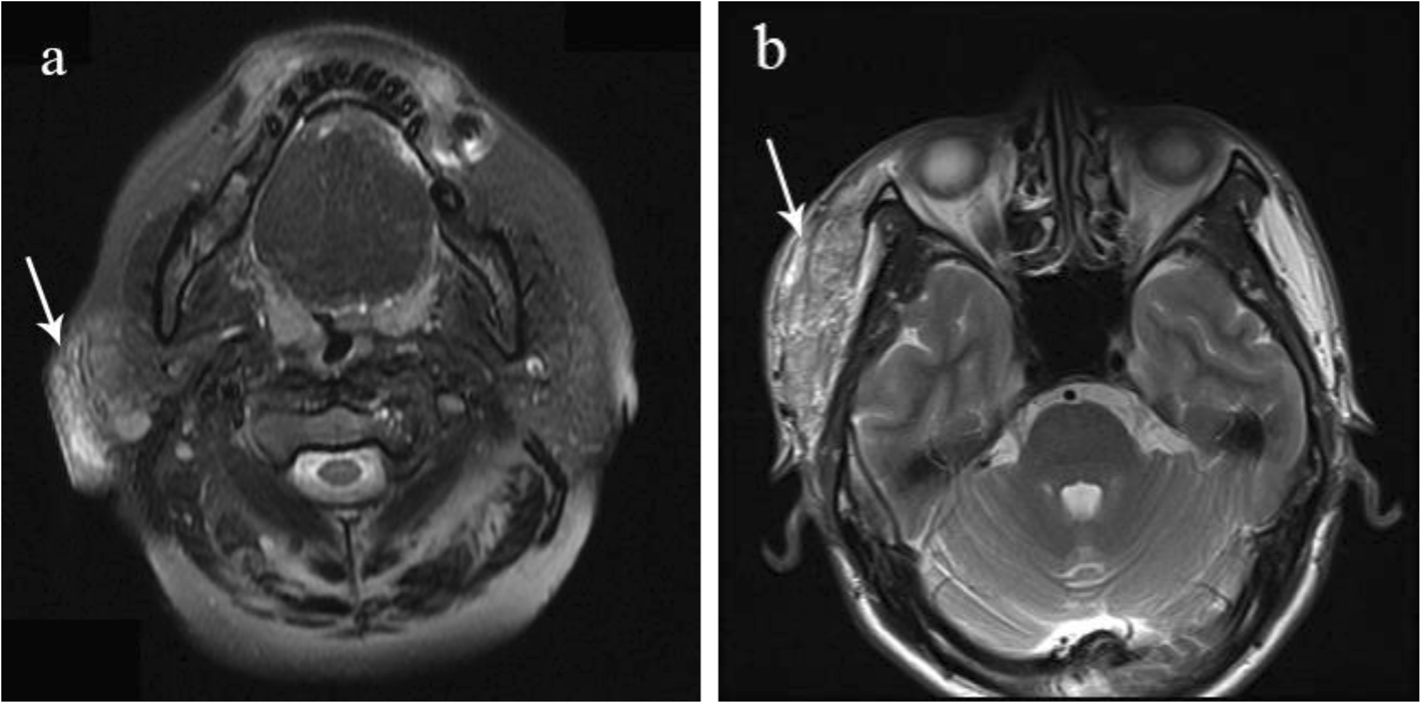
Mass was hyper-intense on axial T2-weighted image on two patients (arrow). **a** A 51-year-old female patient presented with one-year history of a pruritus mass in the right parotid region; **b** A 44-year-old male patient presented with one-year history of a mass with melanin pigmentation in the right temporal region

Seventeen patients underwent imaging examination (Table 2). Among them, 12 underwent ultrasound examination, 8 CT examination, and 5 MRI. At ultrasound, the lesions were found in lymph nodes (25%, 4/16), parotid gland (44%, 7/16), and soft tissues (31%, 5/16). The masses in the lymph and parotid gland exhibited hypoechoic with heterogeneous echotexture and were round or round-like. In contrast, those in the soft tissues exhibited hypoechoic or hyperechoic features (Fig. 2).

**Table 2:** Imaging examination features of 24 patients with Kimura’s disease

Imaging Examination	Ultrasound examination		CT		MRI		
Patients	12		8		5		
Lesions	16		8		5		
Margin							
Undefined	12		7		3		
Well defined	4		1		2		
Characteristics	Hypoechoic	13	Hypo-density	6		T1	T2
					Hypo-intense signal	3	0
	Hyperechoic	3	Iso-density	2	Iso-intense signal	1	0
	Doppler ultrasound				Hyper-intense signal	1	5
			Enhancement				
	High intralesional vascularity	6	Homogeneous	6	Homogeneous	2	
	Low intralesional vascularity	10	Heterogeneous	2	Heterogeneous	3

### Cytology and histopathology examination

**Fig. 3:**
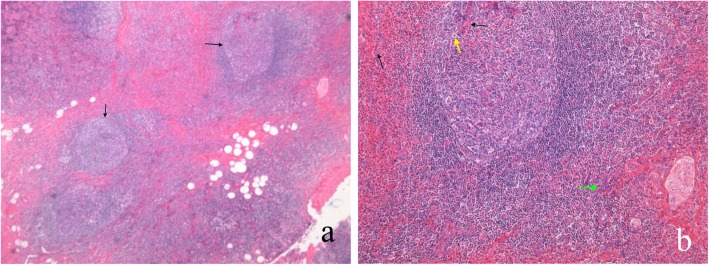
**a** Germinal center hyperplasia (black arrows). **b** Peri and intrafollicular eosinophil infiltrate (black arrows), eosinophil micro-abscess (yellow arrows), and hyperplasia of peripheral post-capillary venules (green arrows)

Out of the 24 patients, 14 were preliminary diagnosed by core-needle biopsy: 6 cases were non-specific lymphadenitis, 2 cases Hodgkin’s lymphoma, 1 tuberculosis of lymph nodes, 1 mixed tumor, 2 suspected to be Langerhans cell histiocytosis, and 2 were diagnosed as KD. All patients were confirmed by surgical specimens. By microscopic analysis, KD mainly involved subcutaneous soft tissue and lymph nodes. The prominent microscopic features were lymphoid tissue presenting germinal center hyperplasia, surrounded by several lobules associated with hyperplastic vascular structures. Lymphoid follicles were composed of germinal centers of variable dimensions, which contained eosinophil infiltrates of various degrees. Eosinophil infiltrated throughout the lesions and focal eosinophil micro-abscesses were observed. Marked vascular hyperplasia was noted, mainly comprising small branched capillaries. Fibrosis was present in almost all cases (Fig. 3). No atypical cells were observed.

### Management and prognosis

A total of 13 patients who had well-defined lesions or definite tumor boundaries underwent SE, and surgical margins were negative. The other patients who had an ill-defined lesion border, multiple lesions, or positive surgical margins underwent SE + OC or SE + R. Follow-up ranged from 6 months to 113 months, and the overall cumulative recurrence rate was 45.8% (11/24). Recurrence rate of SE + R (0/4, 0%) was lower than SE (6/13, 46.2%) and SE + OC (5/7, 71.4%). However, Chi-square test and Fisher’s exact test found it to be statistically insignificant (Table 3). Local recurrence was frequent during the period of steroid dose tapering for SE + OC.

**Table 3:** Local recurrence rates for the three types of treatment modality

Clinical management	No. (patients)	No. (recurrences)	Recurrence rate	Significance
SE + R	4	0	0	
SE	13	6	46.2%	*P*>0.05^a^
SE + OC	7	5	71.4%	*P*>0.05^b^

*SE + R* Surgical resection combined with radiotherapy, *SE* Surgical resection alone, *SE + OC* Surgical resection followed by oral corticosteroids

^a^ *P*-value = 0.237 for the comparison with treatment by SE

^b^ *P*-value = 0.061 for the comparison with treatment by SE + OC

There was no recorded fatality, malignancy resulting from irradiation, or simultaneous occurrence of other systemic diseases in this study. We found no signs of nerve damage or functional impairment and no worsening in the course of other diseases, such as hypertension and diabetes.

## Discussion

Kimura’s disease has been considered as a distinct pathologic entity that was first described in 1937 by Kimm and Szeto in China as “eosinophilic hyperplastic lymphogranuloma”. Subsequently, Kimura et al. described the details of the pathological features of this disease, and this condition has since become widely referred to as Kimura’s disease [3]. The majority of the cases were reported in Asia, mainly in China, Japan, and Southeast Asia, and were usually seen in young men with unilateral involvement [8]. In the present study, the ratio of male to female was 5:1 and unilateral to bilateral was 4:1, which is similar to previous studies [9]. We speculate that sex hormones and genetics may play an important role in the male predominance of KD just like systemic lupus erythematosus which presents with a prominent female predominance.

KD is an immune-mediated inflammatory disorder of unknown etiology. The main presentation is peripheral eosinophilia and elevated serum IgE. In our study, peripheral blood eosinophil count (87.5%) and IgE detection rate (100%) were all at a high level, both of these are of great value for auxiliary diagnosis of KD. It has been speculated that the presentation may be associated with inflammation, endocrinal disorders, autoimmune diseases, parasite infestation, viral infections, and allergies. In our study, we found 6 patients presenting with focal lesions in the head and neck region accompanied by localized inflammation of the head and neck. The concurrence of these diseases might imply a causal relationship with KD. Specifically, a viral or parasitic trigger may alter T-cell immunoregulation or induce an IgE-mediated type-1 hypersensitivity, resulting in the release of eosinophilotrophic cytokines, such as interleukin 5 and interleukin 4. Future studies are needed to test this speculation.

KD follows an indolent clinical course and misdiagnosis is common. The most common clinical feature is asymptomatic unilateral soft-swelling, such as salivary glands and local lymph nodes [3]. In this study, the lesions predominantly involved the salivary gland, preauricular area, and cervical lymph node. The reason may be due to the KD being more likely to involve lymph nodes, while head and neck lymph nodes are numerous. Head and neck lymph nodes first drain to the parotid, periauricular, submandibular, and other upper cervical lymph nodes, then finally into the cervical lymph nodes. KD is frequently associated with pruritus or melanin pigmentation of the overlying skin, probably due to nerve infiltration by lymphocytes and eosinophils [10]. In the present study, 33.3% of the patients presented with pruritus, 20.8% had melanin pigmentation, and 12.5% had coarseness of the affected overlying skin.

Imaging examination has a high detection rate for KD involving the parotid gland and subcutaneous. It is helpful for determining the location and extent of pathological changes and lymph node involvement but has low specificity. Ultrasound examination often shows hypoechoic masses, unclear borders, irregular shape, and uneven internal echo. Consequently, KD is often misdiagnosed as maxillofacial malignant tumors [11]. Shin et al. showed that ultrasound revealed a rich blood flow signal with local subcutaneous hypoechoic and internal hyperechoic features, accompanied by peripheral subcutaneous fat, adjacent lymph node enlargement, and other characteristics, suggesting a high probability of KD [12]. In our study, appearance on radiological findings by different modality such as CT or MRI was variable and not well defined due to variable degrees of vascular proliferation and fibrosis. However, as suggested by Park et al., CT and MRI can be useful for diagnosing KD in the head and neck areas [13]. Characteristic imaging results include multiple ill-defined enhanced masses within and around the parotid gland with associated regional lymphadenopathy.

Pathological examination is the golden standard for the diagnosis of KD. In our study, two patients failed to yield a definite diagnosis based only on Fine-needle aspiration biopsy. Moreover, the accuracy of core-needle biopsy was low (2/14, 14.3%). Therefore, we feel that patients with suspected KD should be confirmed by surgical specimens. The typical characteristics of histopathology of the 24 patients included: Lymphoid follicular hyperplasia and germinal center enlargement; eosinophilic infiltration and accumulation of eosinophilic micro-abscess; and postcapillary and venular hyperplasia, surrounded by circular collagenous fibrous deposition and different extent of fibrosis. Lymph nodes and external lesions have similar pathological manifestations.

The optimal treatment of KD is controversial due to lack of large-scale systemic clinical studies on different treatments for KD. Successful cases have been reported by applying surgery, radiotherapy, and chemotherapy [14]. According to some case reports, different medications, such as cyclosporine, tacrolimus, mycophenolate mofetil, and loratadine [15–17], have been used in the treatment of KD with responses ranging from mild improvement to complete remission or even cure of the disease. As KD is benign, surgical resection does not spread lesions, but helps to reduce lumps and benefits diagnosis. Therefore, surgical excisions have been considered the golden standard of treatment for KD. However, KD typically involves subcutaneous tissue without well-defined boundaries, making it difficult to achieve a negative margin by surgical excision alone and recurrence is possible. In this study, up to 46.2% of recurrence was associated with this modality. A combination of surgery with postoperative intervention seems to be a reasonable approach for treatment of KD. Nakahara et al. reported that steroid therapy can control lesions, lymphadenopathy, and nephrotic syndrome in KD, but local recurrence occurred frequently during the period of steroid dose tapering [18], which is consistent with our findings. Radiotherapy has been used to treat recurrent or persistent lesions with better rates for local control, but the carcinogenic effects of radiotherapy along with the benign nature of the disease limits its use as the primary modality. However, Ye et al. stated that surgical resection combined with low-dose postoperative radiotherapy for treatment of KD achieved the lowest local recurrence rate [14]. In this study, the recurrence rate following surgical excision combined with low-dose radiotherapy was lower than that of either surgical excision alone or surgical resection followed by oral corticosteroids. However, Chi-square test and Fisher’s exact test found this to be statistically insignificant, but this may have been caused by small number of patients. More cases are needed to confirm this conclusion.

Relapse is an important characteristic of KD. In this study, the overall recurrence rate was 46.8%. Among the 11 patients with recurrence, the ratio of male to female was 2:1; involving patients in all age groups and the most common recurrence site was in situ. The neck and parotid gland were the most likely to relapse. For relapses, the treatment as described above was still effective.

## Conclusions

In summary, for patients with subcutaneous painless soft tissue swelling in the head and neck, a high possibility of KD should be considered if peripheral blood eosinophils increase, especially when IgE levels increase in the serum. Pathological examination is the gold standard for diagnosis of KD which should be confirmed by surgical specimens. KD has a high recurrence rate but good prognosis. Surgical excision combined with low-dose radiation therapy should be carried out for KD to reduce local recurrence.

## Data Availability

The datasets used and analysed during the current study are available from the corresponding author on reasonable request.

## Abbreviations

KD: Kimura’s disease
SE + OC: Surgical resection followed by oral corticosteroids
SE + R: Surgical resection combined with radiotherapy
SE: Surgical resection alone
